# A Method for Assessing the Retention of Trace Elements in Human Body Using Neural Network Technology

**DOI:** 10.1155/2017/3471616

**Published:** 2017-07-16

**Authors:** Yulia Tunakova, Svetlana Novikova, Aligejdar Ragimov, Rashat Faizullin, Vsevolod Valiev

**Affiliations:** ^1^Kazan National Research Technical University named after A. N. Tupolev (KAI), Kazan, Russia; ^2^The I.M. Sechenov First Moscow State Medical University, Moscow, Russia; ^3^Institute of Fundamental Medicine and Biology, Kazan Federal University, Kazan, Russia; ^4^Institute of Problems of Ecology and Subsoil Resources of the Academy of Sciences of Tatarstan, Kazan, Russia

## Abstract

Models that describe the trace element status formation in the human organism are essential for a correction of micromineral (trace elements) deficiency. A direct trace element retention assessment in the body is difficult due to the many internal mechanisms. The trace element retention is determined by the amount and the ratio of incoming and excreted substance. So, the concentration of trace elements in drinking water characterizes the intake, whereas the element concentration in urine characterizes the excretion. This system can be interpreted as three interrelated elements that are in equilibrium. Since many relationships in the system are not known, the use of standard mathematical models is difficult. The artificial neural network use is suitable for constructing a model in the best way because it can take into account all dependencies in the system implicitly and process inaccurate and incomplete data. We created several neural network models to describe the retentions of trace elements in the human body. On the model basis, we can calculate the microelement levels in the body, knowing the trace element levels in drinking water and urine. These results can be used in health care to provide the population with safe drinking water.

## 1. Introduction

It is known that there is a complex dependence between water and food intake and trace element retention with the formation of their individual statuses, and this dependence has generally a nonlinear nature. Therefore, linear modelling methods in multivariate regression tasks are not able to describe with sufficient precision the whole range of relations among significant factors appearing in the models developed for the human body [1–8].

A number of studies [9–18] assert that trace elements enter the body primarily through water and food intake, then they are carried by the blood by binding them to specific proteins; however, a certain portion of them (which is different for different elements) is present in the blood in an ionized form. The kidneys regulate the trace elements balance by excreting them in the urine. At the same time, the proportion of trace element forms not bound to proteins drastically increases in the setting of an excessive admission from the outside due to homeostatic limits to the possible presence of transport proteins and, as a consequence, lack of reserves for binding. Under these conditions, the excretory function of the kidneys grows and the concentration of elements in urine increases.

The modelling of the dynamics of trace elements concentration in the serum as well as the process of excretion of minerals in the urine are an important stage that characterizes the processes associated with trace element retention. Thus, this research is aimed at creating models that adequately reflect the balance of the essential elements, as well as the processes of their intake, excretion, and, especially, retention in the body.

The possibilities of neural network methods, which automatically take into account both explicit and implicit dependencies, exist among initial data [19–21]. In addition, neural networks, in contrast to traditional modelling methods, allow for using incomplete and inaccurate input data and are able to reflect nonlinear dependencies and choose the right correction coefficients [22]. However, the use of neural networks is restricted to simple networks of direct distribution. Moreover, scientific publications in the recent years do not contain information regarding the use of neural networks for the assessment and correction of the balance of trace elements in the body. The use of neural networks of “multilayer perceptron” type, self-organizing maps (Kohonen maps or Kohonen networks) and probabilistic neural networks, as well as hybrid and cascade networks (multilayer neural network experts, neural network cascades, etc.), allows for reducing calculation errors from tens to a few percent and even to tenths of percent.

## 2. Materials and Methods

In order to model the processes of retention of trace elements in the body, we analyzed more than 2000 samples of dynamic internal media of children and adolescents living in the city of Kazan (Russia) and more than 750 samples of drinking water consumed by them to assess the concentration of the most common trace elements (Zn, Cu, Fe, Pb, Cr, and Sr).

The blood samples tested in the research were centrifuged for 15 minutes at 3000 rev/min. The calibration solutions (stock and working) were prepared based on the State Standard Samples by the standard method. To measure the metal concentrations in the blood, we previously diluted the serum with bidistilled water at 1 : 2 ratio (to detect Zn, Cu, and Fe) or in a TCA filtrate (in the case of Pb, Cr, and Sr). To obtain the TCA filtrate, we hydrolyzed whey proteins in hydrochloric acid (reagent grade), adding 0,75 ml of 1,5% HCl solution to 1,5 ml of serum and incubating for 1 hour at 37°C. After hydrolysis, the proteins were precipitated by 0,75 ml of 20% TCA (trichloroacetic acid), with a final dilution at 1 : 2 ratio, and after 1 h were centrifuged for 10 minutes at 1500 rev/min. The supernatant fluid (TCA filtrate) was collected for analysis.

In those cases when the concentration of a certain trace element was below the detection level and could not be detected directly in the TCA filtrate, we used the concentration/extraction method: added 0,5 ml of 2% sodium diethyldithiocarbamate solution and 2 drops of TRITON-X-100 detergent to 2,5 ml of serum and vigorously shook the mixture for 10 seconds. Then, we let the mixture settle for 10 minutes, added 1,5 ml of butyl acetate, shook it for 1 minute, centrifuged it, and analyzed the extract. This way, we managed to reduce the detection level by a factor of 1,5 for chrome and by a factor of 2,5 for strontium and lead.

In order to determine the trace elements in urine, we collected daily urine samples and examined the concentrations of metals in them by direct analysis.

It is known that the ionized forms of trace elements entering the body with consumed water are effectively assimilated. Trace element salts dissociated in water are characterized by a high biological activity; the adsorption of these salts in the gastrointestinal tract is very quick and complete. Therefore, the fraction of trace elements entering the body in a dissolved form must manifest itself in some way in the change of their concentrations in the serum [5].

During preparation of drinking water samples, we evaporated 1 litre of water in a water bath and then dissolved the solid residue in 50 ml of 1 N nitric acid (reagent grade). The obtained aliquot part was analyzed by the AAS method.

As the analytical method for determining trace elements in the examined media, we chose the AAS method, since it is known as one of the most selective and reproducible methods and is recognized for its high selectivity and speed of execution, which becomes a very important factor when performing a research at a population level. This method is especially adequate for the analysis of solutions, since in this case the dissociation of the analyzed substance into atoms can be achieved by heating in a Bunsen burner. The detection of trace elements in a highly oxygenated air-acetylene flame is highly selective and characterizes itself by an insignificant influence of the sample composition on the analysis result. The primary statistical processing of the results was performed using the software package “STATISTICA 6.” We evaluated the confidence intervals, variances, quartiles, the normality of distributions, and the statistical significance (*t*-test). The significance of the results was determined using a 95% confidence interval (*p* < 0, 05).

MLP-type neural networks were chosen as the paradigm of the regression model [23]. The structure of this type of neural networks is defined empirically and is determined by the complexity of the information contained in the data. For training the neural networks, we used a network reduction method based on multiobjective optimization [24].

## 3. Results

A wide concentration gradient was detected for all trace elements in the tested media. Results from previous experiments show that knowing the content of trace elements in drinking water is not enough to build adequate models describing how these elements enter the blood and subsequently are excreted in the urine. For this reason, we decided to supplement the model with information on some physiological characteristics of the human organism. The height and weight of the tested individual were taken as anthropometric factors affecting directly the processes of accumulation of metals in the organism and their excretion from it. These factors define a key morphometric parameter widely used in toxicology and human physiology, and known as the body surface area (1), which characterizes indirectly the length of the circulatory system:

(1) $$\begin{array}{c} S_{body}=\sqrt{\frac{P\times B}{3600}}, \end{array}$$

where *S*_body_ is the body surface area, measured in m^2^, *Р* is the height, measured in cm, and *В* is the weight, measured in kg.

In order to increase the accuracy of the calculation of the concentration of ionized trace elements in the urine, we introduced a characteristic determined by the excretory function of the kidneys, namely, the “daily diuresis,” which is the total volume of urine (in ml) produced by the human organism per day.

Thus, to calculate the retention level, we used data tuples of the following form: “[Concentration of trace elements in drinking water] & [Concentration of trace elements in serum] & [Concentration of trace elements in urine] − [Retention level in the body]”. The input parameter, “Concentration of trace elements in drinking water” was determined by direct measurement, whereas the parameters “Concentration of trace elements in serum” and “Concentration of trace elements in urine” were computed in a cascade-like manner, on the basis of separated neural network models.

The model for assessing the retention level is based on a fuzzy inference system [25], since the models of this kind take into account the blurred boundaries of the notions of “low” (coded with 0) and “high” (coded with 1) for the trace element levels in water, blood, and urine, and also reflects the nonlinear nature of the dependence of the factors that determine the retention. The linguistic values are correlated with the quantitative values of the concentration of trace elements in drinking water, blood and urine (mg/l) by means of Gaussian membership functions with centres computed as the boundary between the first (lower) and the third (upper) quartiles on the basis of a series of outdoor measurements (see Table 1).

## 4. Discussion

The following reasoning allows to determine the character and values of the output parameter “Retention level.” After analyzing the regression coefficients of the obtained linear models describing the correlation between the concentrations of trace elements in drinking water, serum, and urine, it may be noted that they are in the ratios (“Water” : “Blood” : “Urine”) 5 : 2 : 3. In particular, if we consider the regression of the ratios of the indices of excess rate, which are the values, reduced with respect to the median of the series, of each element of the sample (*R*_retention_, *R*_water_, *R*_blood_, *R*_urine_), then it has the form

(2) $$\begin{array}{c} R_{retention}=0,327+0,52\times R_{water}+0,19\times R_{blood}-0,33\times R_{urine}. \end{array}$$

If we take a conventional unit as maximum intensity of the retention, then the weighting coefficients of the factors of the sequence “Water” => “Blood” => “Urine” are distributed as follows:

- a high level of trace elements in blood (coded with 1) corresponds to a weight of 0,2, whereas a low level of trace elements in blood (coded with 0) corresponds to a weight of −0,2;
- a high level of trace elements in drinking water (coded with 1) corresponds to a weight of 0,5, whereas a low level of trace elements in drinking water (coded with 0) corresponds to a weight of −0,5;
- a high level of trace elements in urine (coded with 1) corresponds to a weight of 0,3, whereas a low level of trace elements in urine (coded with 0) corresponds to a weight of −0,3.

As we see, the intensity of retention varies from −1, in cases of excessive excretion, to +1, when the retention level is maximal. When the retention level equals to 0, no concentration changes occur in the body, and the system stays in a state of equilibrium.

Thus, the rules of inference take the following meaning and describe the intensity and direction of the retention processes (“Water” : “Blood” : “Urine”):

1. **IF** (Concentration of trace elements in drinking water = “low”) **AND** (Concentration of trace elements in serum = “low”) **AND** (Concentration of trace elements in urine = “low”) **THEN** Retention level = −0,5 − 0,2 + 0,3 = −0,4, so it is “moderately reduced”.
2. **IF** (Concentration of trace elements in drinking water = “low”) **AND** (Concentration of trace elements in serum = “low”) **AND** (Concentration of trace elements in urine = “high”) **THEN** Retention level = −0,5–0,2–0,3 = −1,0, so it is “minimal”.
3. ... , and so on.

Considering the adopted codes, the basic set of rules can be represented as seen in Table 2.

This distribution gives a dynamic characteristic of the retention levels of trace elements in the body, with an emphasis on the state balance.

There are two fuzzy inference systems that can be reasonably considered as suitable for solving this problem: a Mamdani system with a fuzzy and/or defuzzification of the output and a Takagi-Sugeno system with defuzzification of the linear output. In the case of the Mamdani inference system, the quantitative expressions of the linguistic values of the output variable “Retention level” are either the centre points of the output Gaussian membership functions or singletons. In the case of the Takagi-Sugeno fuzzy system, the quantitative values obtained are the constant terms (*y*-intercepts) of the linear combinations of the inputs having zero coefficients [26].

**Figure 1:**
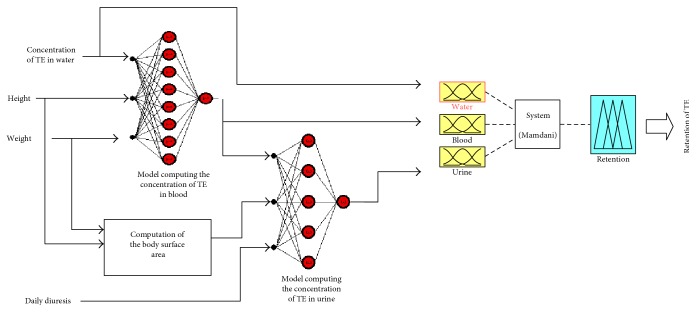
Structure of the model assessing the retention of trace elements.

We constructed a hybrid intelligent model consisting of two cascade-coupled neural networks [27] as a practical realization of the proposed approach to determining concentrations of metals in serum and urine, respectively, based on data on the concentrations of trace elements in drinking water and on the physical anthropological characteristics of the tested individuals. The third element of the model is a Mamdani inference system with a definition of the Gaussian membership functions for the output parameter (retention level) based on fuzzy initial data on the concentrations of trace elements in drinking water, serum, and urine obtained in the previous stages. The model assessing the retention of any of the tested trace elements (TE) features the structure shown in Figure 1.

The practical approval of the trace elements retention model was carried out in a test group of children and adolescents. Tables 3–5 provides some results of experiments in the case of zinc.

**Figure 2:**
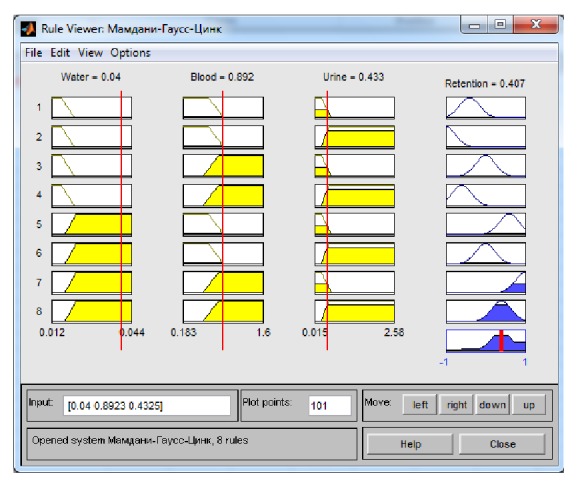
Results of the data processing for Table 3 using the Mamdani system.

As a result of cascade modelling, we obtained the following values for zinc concentrations in serum and urine: 0,892 mg/l and 0,433 mg/l, respectively (Table 3). These values were input to the Mamdani system. The results of the data processing are shown in Figure 2.

As a result, the value of zinc retention was found to be 0,407 mg/l, which corresponds to a moderately high level.

**Figure 3:**
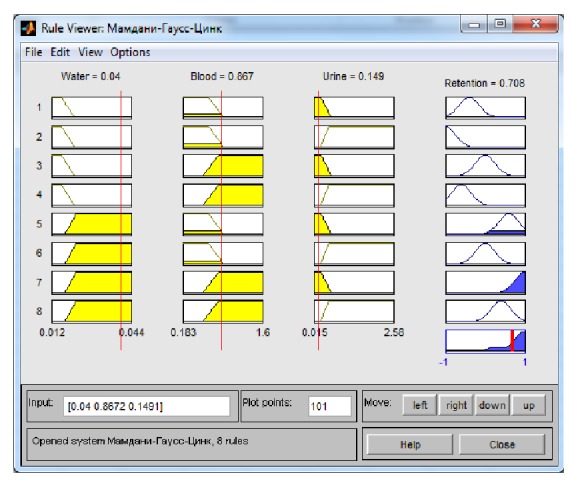
Results of the data processing for Table 4 using the Mamdani system.

As a result of cascade modelling, the following values were obtained for zinc concentrations in serum and urine: 0,867 mg/l and 0 mg/l, respectively (Table 4). The results of the data processing are shown in Figure 3.

The value obtained for zinc retention was 0,708 mg/l, corresponding to a high level.

**Figure 4:**
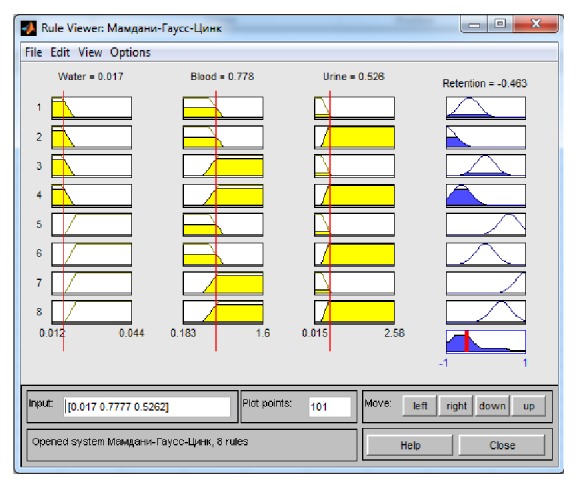
Results of the data processing for Table 5 using the Mamdani system.

We obtained, as a result of cascade modelling, the following values for zinc concentrations in serum and urine: 0,778 mg/l and 0,527 mg/l, respectively (Table 5). The data processing results are shown in Figure 4.

The negative value obtained for the zinc retention (−0,463 mg/l) is an evidence of excessive excretion of zinc and corresponds to a moderately low level.

## 5. Conclusions

The method we propose here for assessing and expressing quantitatively the retention of trace elements in the human body is based on a cascade hybrid intelligent system that recommends itself by its high degree of accuracy and reliability. This method does not require expensive laboratory studies and allows for assessing the value of the retention in the body using easily accessible information. The simplified structure of the neural network regression model (its reduced number of inputs) provides sufficient accuracy, and the reduction of the neural networks increases the adequacy of the models.

This method for assessing the retention can be used in the subsequent determination of the balance of trace elements in the human body and the choice of an appropriate method for imbalance correction, both at individual and population levels.

## Figures and Tables

**Table 1:** Correlation between linguistic and quantitative values of the input parameters.

Input parameter	Trace element	Linguistic value	Centre value of the membership function
Trace elements concentration in drinking water	Zinc	Low level	0,016
		High level	0,022
	Chrome	Low level	0,0012
		High level	0,0045
	Iron	Low level	0,0735
		High level	0,1
	Strontium	Low level	0,107
		High level	0,17775
	Copper	Low level	0,0012
		High level	0,0018
	Lead	Low level	0,012
		High level	0,0165

Trace elements concentration in serum	Zinc	Low level	0,6355
		High level	0,8275
	Chrome	Low level	0,04
		High level	0,08525
	Iron	Low level	1,1375
		High level	1,9635
	Strontium	Low level	0,08925
		High level	0,156
	Copper	Low level	0,715
		High level	0,99275
	Lead	Low level	0,0475
		High level	0,079

Trace elements concentration in urine	Zinc	Low level	0,239
		High level	0,4895
	Chrome	Low level	0,012
		High level	0,028
	Iron	Low level	0,0745
		High level	0,2195
	Strontium	Low level	0,087
		High level	0,222
	Copper	Low level	0,022
		High level	0,084
	Lead	Low level	0,028
		High level	0,055

**Table 2:** Table of rules of inference.

Trace elements concentration in drinking water	Trace elements concentration in serum	Trace elements concentration in urine	Retention level	
			Value	Linguistic value
0	0	0	–0,4	Moderately low
0	0	1	−1	Minimal
0	1	0	0	Equilibrium state
0	1	1	−0,6	Low
1	0	0	0,6	High
1	0	1	0	Equilibrium state
1	1	0	1	Maximal
1	1	1	0,4	Moderately high

**Table 3:** 

Weight (kg)	Height (cm)	Body surface area (m^2^)	Daily diuresis (ml)	Zinc concentration in drinking water (mg/l)
40,15	164	1,352	750	0,04

**Table 4:** 

Weight (kg)	Height (cm)	Body surface area (m^2^)	Daily diuresis (ml)	Zinc concentration in drinking water (mg/l)
41,8	170	1,450	1300	0,04

**Table 5:** 

Weight (kg)	Height (cm)	Body surface area (m^2^)	Daily diuresis (ml)	Zinc concentration in drinking water (mg/l)
57	159	1,587	720	0,017

## References

[1] Coelho P., Costa S., Silva S., Walter A. (2012). Metal(loid) levels in biological matrices from human populations exposed to mining contamination-Panasqueira mine (Portugal). *Journal of Toxicology and Environmental Health. Part A*.

[2] Schmidl D., Hug S., Li W. B., Greiter M. B., Theis F. J. (2012). Bayesian model selection validates a biokinetic model for zirconium processing in humans. *BMC Systems Biology*.

[3] Konzen K., Miller S., Brey R. (2015). Proposed modification to the plutonium systemic model. *Health Physics*.

[4] Unice K. M., Kerger B. D., Paustenbach D. J., Finley B. L., Tvermoes B. E. (2014). Refined biokinetic model for humans exposed to cobalt dietary supplements and other sources of systemic cobalt exposure. *Chemico-Biological Interactions*.

[5] Friedrich M., Kuchlewska M. (2013). Assessing the effect, on animal model, of mixture of food additives, on the water balance. *Acta Scientiarum Polonorum. Technologia Alimentaria*.

[6] Bitto A., Horvath A., Sarkany E. (1997). Monitoring of blood lead levels in Hungary. *Central European Journal of Public Health*.

[7] Aggett P. J. (1985). Physiology and metabolism of essential trace elements. *Clinics in Endocrinology and Metabolism*.

[8] Malinovsky G., Yarmoshenko I., Zhukovsky M., Starichenko V., Modorov M. (2013). Strontium biokinetic model for mouse-like rodent. *Journal of Environmental Radioactivity*.

[9] Hanson N., Stark J. D. (2012). Comparison of population level and individual level endpoints to evaluate ecological risk of chemicals. *Environmental Science & Technology*.

[10] Ozmen H., Akarsu S., Polat F., Cukurovali A. (2013). The levels of calcium and magnesium, and of selected trace elements, in whole blood and scalp hair of children with growth retardation. *Iranian Journal of Pediatrics*.

[11] Chalvatzaki E., Lazaridis M. (2015). Development and application of a dosimetry model (ExDoM2) for calculating internal dose of specific particle-bound metals in the human body. *Inhalation Toxicology*.

[12] Leggett R. W. (2012). A biokinetic model for zinc for use in radiation protection. *The Science of the Total Environment*.

[13] Rainone F., Arcidiacono T., Terranegra A. (2011). Calcium sensing receptor and renal mineral ion transport. *Journal of Endocrinological Investigation*.

[14] Klaassen C. D., Liu J., Diwan B. A. (2009). Metallothionein protection of cadmium toxicity. *Toxicology and Applied Pharmacology*.

[15] Leggett R. W. (2008). The biokinetics of inorganic cobalt in the human body. *The Science of the Total Environment*.

[16] Kiss S. A., Forster T., Dongó A. (2004). Absorption and effect of the magnesium content of a mineral water in the human body. *Journal of the American College of Nutrition*.

[17] Kiss S. A., Forster T., Dongó A. (2004). Absorption and effect of the magnesium content of a mineral water in the human body. *Journal of the American College of Nutrition*.

[18] Wesseling-Perry K., Salusky I. B. (2013). Chronic kidney disease: mineral and bone disorder in children. *Seminars in Nephrology*.

[19] Lek S., Guegan J.-F. (2000). *Artificial Neuronal Networks, Application to Ecology and Evolution*.

[20] Zeng X., Li Y., He R. (2015). Predictability of the loop current variation and eddy shedding process in the Gulf of Mexico using an artificial neural network approach. *Journal of Atmospheric and Oceanic Technology*.

[21] Zeng X., Li Y., He R., Yin Y. (2015). Clustering of loop current patterns based on the satellite-observed sea surface height and self-organizing map. *Remote Sensing Letters*.

[22] Zadeh L. A. (1996). Fuzzy sets, fuzzy logic, and fuzzy systems fuzzy sets. *Advances in Fuzzy Systems - Applications and Theory*.

[23] Hornik K., Stinchcombe M., White H. (1989). Multilayer feedforward networks are universal approximators. *Neural Networks*.

[24] Schmidhuber J. (2015). Deep learning in neural networks: an overview. *Neural Networks*.

[25] Margaret A. (1998). Boden creativity and artificial intelligence. *Artificial Intelligence*.

[26] Li B., Zhu Y. (2015). Uncertain linear systems. *Fuzzy Optimization and Decision Making*.

[27] Granitto P. M., Verdes P. F., Ceccatto H. A. (2005). Neural network ensembles: evaluation of aggregation algorithms. *Artificial Intelligence*.

